# The role of PD-1 signaling in health and immune-related diseases

**DOI:** 10.3389/fimmu.2023.1163633

**Published:** 2023-05-16

**Authors:** Ru-Yue Chen, Yun Zhu, Yun-Yan Shen, Qin-Ying Xu, Han-Yun Tang, Ning-Xun Cui, Lu Jiang, Xiao-Mei Dai, Wei-Qing Chen, Qiang Lin, Xiao-Zhong Li

**Affiliations:** Department of Nephrology and Immunology, Children’s Hospital of Soochow University, Suzhou, Jiangsu, China

**Keywords:** programmed cell death 1 receptor, immune checkpoint proteins, immune tolerance, tumor microenvironment, autoimmune diseases, immunotherapy

## Abstract

Programmed cell death 1 receptor (PD-1) and its ligands constitute an inhibitory pathway to mediate the mechanism of immune tolerance and provide immune homeostasis. Significantly, the binding partners of PD-1 and its associated ligands are diverse, which facilitates immunosuppression in cooperation with other immune checkpoint proteins. Accumulating evidence has demonstrated the important immunosuppressive role of the PD-1 axis in the tumor microenvironment and in autoimmune diseases. In addition, PD-1 blockades have been approved to treat various cancers, including solid tumors and hematological malignancies. Here, we provide a comprehensive review of the PD-1 pathway, focusing on the structure and expression of PD-1, programmed cell death 1 ligand 1 (PD-L1), and programmed cell death 1 ligand 2 (PD-L2); the diverse biological functions of PD-1 signaling in health and immune-related diseases (including tumor immunity, autoimmunity, infectious immunity, transplantation immunity, allergy and immune privilege); and immune-related adverse events related to PD-1 and PD-L1 inhibitors.

## 1 Introduction

**Figure 1:**
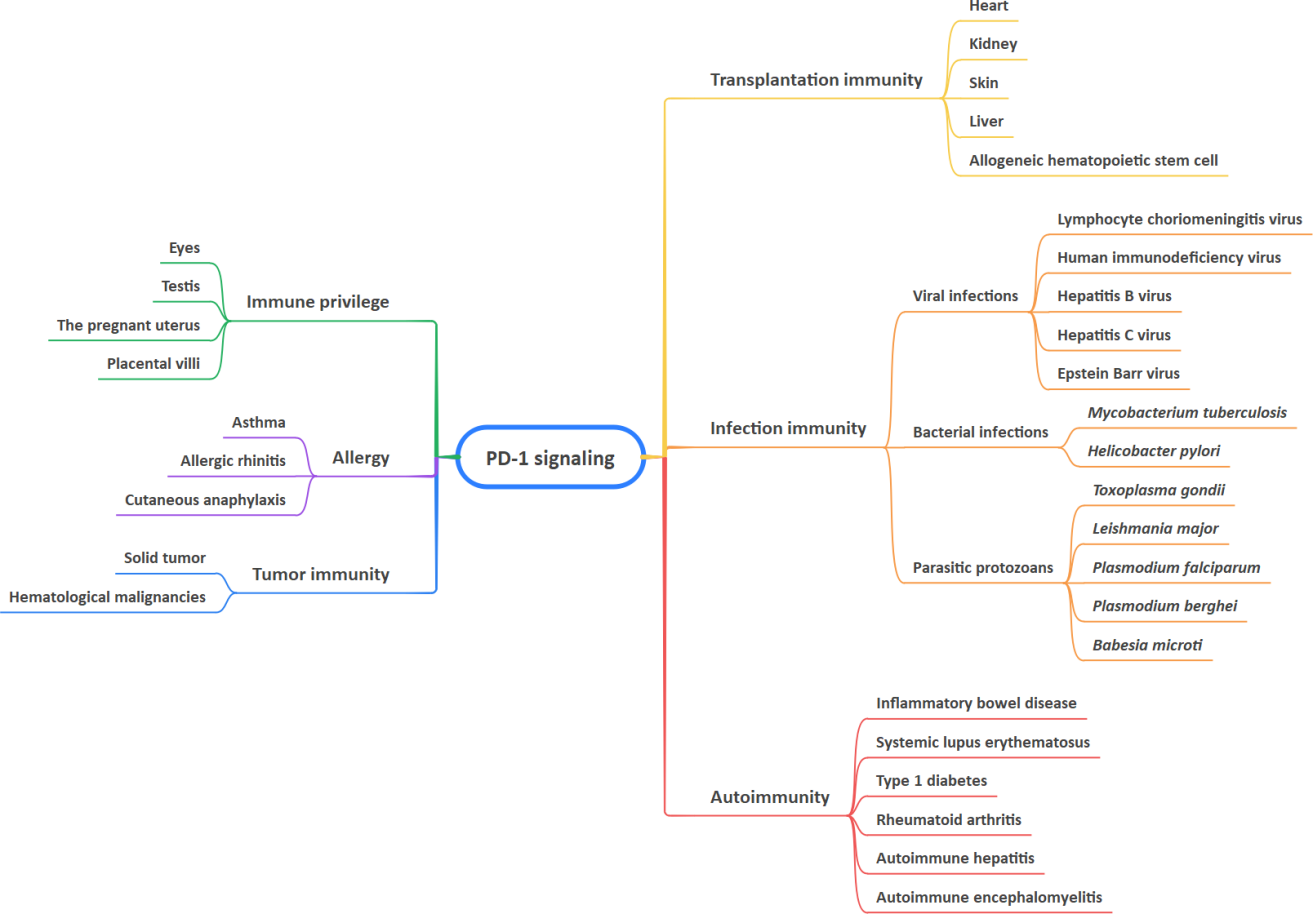
Involvement of PD-1 signaling in health and immune-related diseases.

Recent years have seen a rapid expansion of our knowledge of immune regulation. T-cell activation is a key step in the initiation and modulation of the immune response (1). The activation of T cells relies mainly on a two-signal model. The first signal confers specific recognition of cognate antigenic peptides presented by major histocompatibility complex (MHC) molecules, which triggers T cell receptor (TCR) signaling. The second signal comprises co-stimulatory and co-inhibitory signals, which modulate TCR signaling positively or negatively to direct T cell function (1–3). A group of inhibitory or stimulatory molecules expressed on immune cells, antigen-presenting cells (APCs), tumor cells, or other types of cells are regarded as immune checkpoints, including programmed cell death-1 (PD-1), cytotoxic T lymphocyte-associated antigen-4 (CTLA-4), cluster of differentiation 28 (CD28), cluster of differentiation 80 (CD80), galectin-9 (Gal-9), and T cell immunoglobulin and mucin domain 3 (TIM-3) (4–7). Immune checkpoint pathways are defined as receptor-ligand pairs that exert inhibitory or stimulatory effects on immune responses. It is noteworthy that the binding partners of the receptor and its associated ligands are diverse, which facilitates immunosuppression in cooperation with other immune checkpoint proteins (6–8). To date, increasing numbers of studies have indicated that PD-1 and its ligands are involved in maintaining immune-related diseases, particularly tumor-associated biological features (9–13). This article provides a review of the role of PD-1 signaling in health and immune-related diseases, including tumor immunity, autoimmunity, infection immunity, transplantation immunity, allergy, and immune privilege, as well as immune-related adverse events (irAEs) of anti-PD-1 and anti-PD-L1 drugs (**Figure 1**).

## 2 Structure and expression of the PD-1 pathway

### 2.1 PD-1

Programmed cell death 1 protein (PD-1, also known as PDCD1 and CD279) is a 50–55-kDa type I transmembrane protein comprising 288 amino acid residues, which includes an immunoglobulin (Ig) superfamily domain, a 20 amino acid stalk, a transmembrane domain, an intracellular domain of approximately 95 residues containing an immunoreceptor tyrosine-based inhibitory motif (ITIM), and an immunoreceptor tyrosine-based switch motif (ITSM) (4, 13, 14). PD-1 belongs to the CD28 superfamily and is encoded by the *PDCD1* gene on human chromosome 2 (4). PD-1 is 15% similar to the amino acid sequence of CD28, 20% similar to CTLA-4, and 13% similar to inducible co-stimulatory molecule (ICOS) (13, 15). PD-1 is a representative immunosuppressive checkpoint and is mainly expressed in activated T lymphocytes, B lymphocytes, natural killer cells, macrophages, dendritic cells, monocytes, and myeloid cells (11). PD-1 expression also occurs in immune-privileged sites, such as the cornea, retina, and iris-ciliary body, and its expression is wider than the restricted expression of other CD28 family members on T cells, resulting in a broader spectrum of immune responses (14). PD-1 expression might be triggered by transcription factors, such as nuclear factor of activated T cells (NFAT), Forkhead box protein (FOX), and interferon regulatory factor 9 (IRF9) (11, 13).

**Figure 2:**
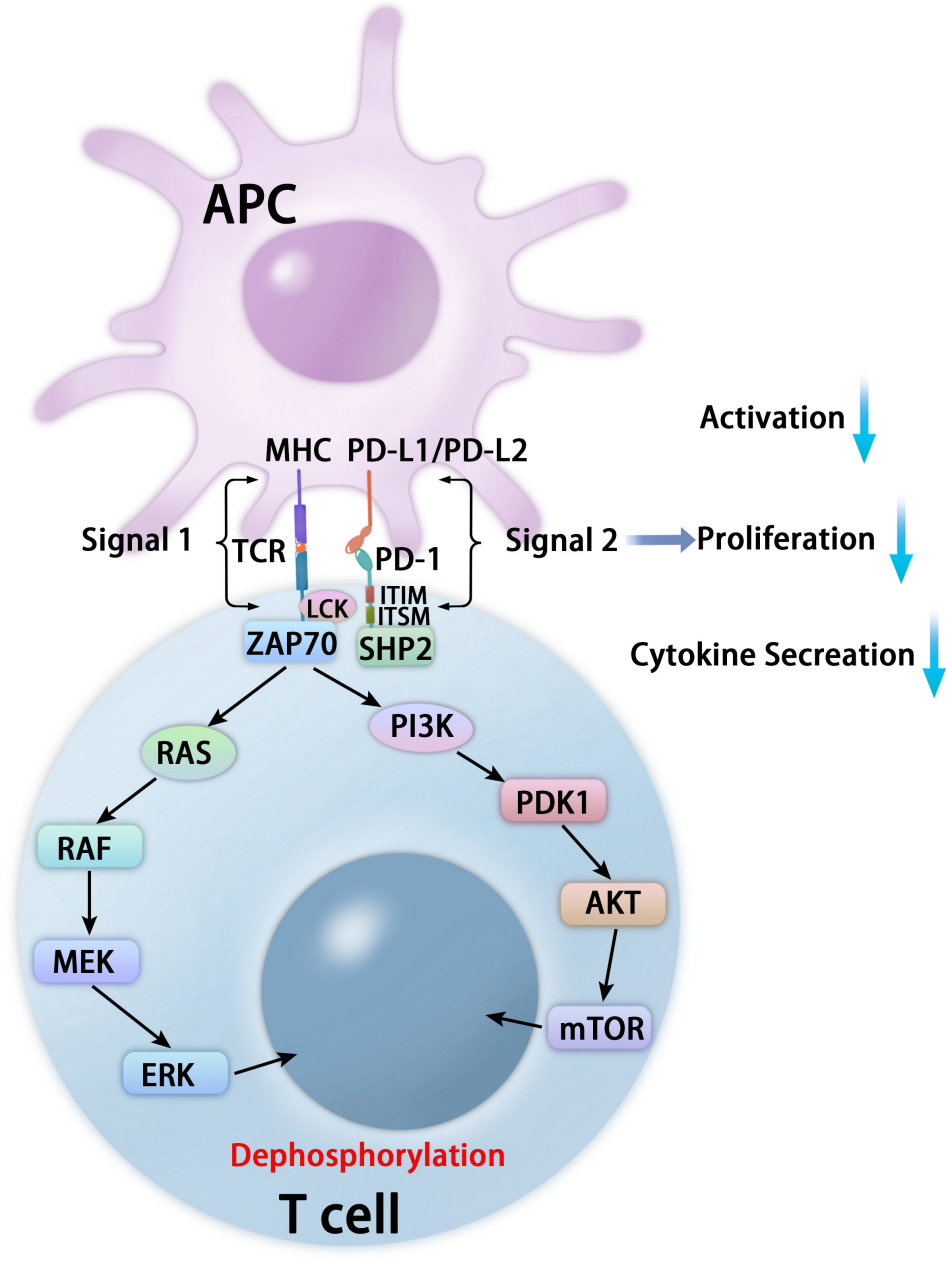
Mechanisms of PD-1-mediated inhibition in T cells. After interacting with PD-L1 or PD-L2, PD-1 recruits the phosphatase SHP-2 in proximity to TCR, which attenuates key TCR proximal signaling, including the PI3K-PDK1-AKT-mTOR pathway and the RAS-RAF-MEK-ERK pathway.

PD-1 binds to two classical ligands: PD-L1 and PD-L2, leading to inhibition of T cell proliferation, activation, cytokine production, altered metabolism, cytotoxic T lymphocytes (CTLs) killer functions, and eventual death of activated T cells (16–18). The inhibitory function depends on interaction with phosphatase SHP-2 (19). PD-1 contains inhibitory motifs, including ITIM and ITSM, and the interaction of the SHP-2 SH2 domains with PD-1 ITSM induces PD-1 dimerization and SHP-2 activation (19, 20). After interacting with its ligands, PD-1 is activated and recruits the phosphatase SHP-2 in proximity to TCRs, which dephosphorylates critical protein molecules for TCR signaling and affects downstream signaling pathways, including the phosphoinositide 3-kinase (PI3K)-phosphoinositide-dependent kinase 1 (PDK1)-AKT-mammalian target of rapamycin (mTOR) pathway, RAS-RAF-MEK (mitogen-activated protein kinase kinase or MAPKK)-extracellular-signal-regulated kinase (ERK) pathway, and Janus kinases (JAKs)-signal transducers and activators of transcription (STAT) pathways (17, 18, 20, 21) (**Figure 2**).

**Figure 3:**
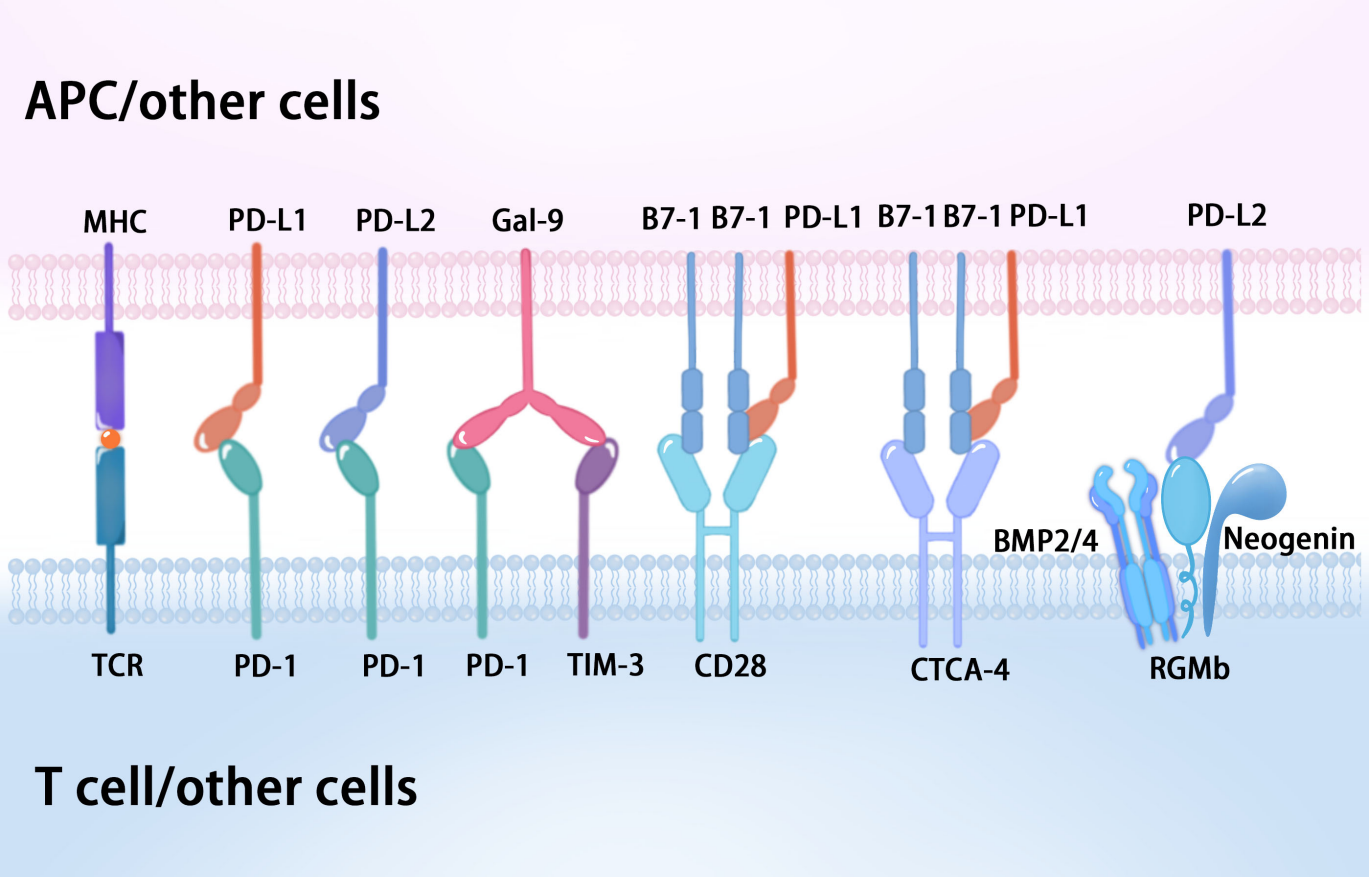
The diverse binding partners of PD-1 and its associated ligands.

Recent evidence has shown that Gal-9 can also interact with PD-1 on the T cell surface, which contributes to the persistence of PD-1+TIM-3+ T cells and attenuates Gal-9/TIM-3-induced cell death (22). Gal-9 is a member of the lectin family of proteins, which acts through receptors such as TIM-3, V-domain Ig suppressor of T cell activation (VISTA), and PD-1 in CTLs (23). Gal-9:PD-1 binding is highly selective and primarily mediated by the C-terminal carbohydrate-recognition domain (CRD) of Gal-9 and the N116-linked glycan of PD-1, which does not affect PD-1 binding to its cognate ligand PD-L1 or the PD-1 therapeutic antibodies pembrolizumab and nivolumab (22) (**Figure 3**). At present, the B7 family comprises ten members: B7-1 (CD80), B7-2 (CD86), B7-H1 (PD-L1), B7-DC (PD-L2), B7-H2, B7-H3, B7-H4, B7-H5 (VISTA), B7-H6, and B7-H7 (24, 25). VISTA is also known as PD-1 homolog (PD-1H), and structural analysis showed that the IgV domain of VISTA shares sequence homology with both CD28 and the B7 family, while the full length VISTA shows the highest identity with PD-1 (26, 27). Significantly, Gal-9 can induce leakage of the proteolytic enzyme granzyme B from the intracellular granules of CTLs, leading to their programmed death via VISTA and TIM-3, which prevents them from interacting with PD-1 (23). Furthermore, human VISTA has two confirmed binding partners with immunosuppressive functions, P-selectin glycoprotein ligand 1 (PSGL-1) and V-set and Ig domain-containing 3 (VSIG3), as well as a less well confirmed receptor, VSIG8 (28). VISTA activity imposes quiescence on mammalian myeloid and naïve T cells, and inhibits T cell activation and cytokine production, which suggests VISTA as a promising target for combination cancer immunotherapy (28).

### 2.2 PD-L1

Programmed cell death 1 ligand 1 (PD-L1, also known as B7-H1 or CD274) consists of 290 amino acid residues, and is a 33-kDa type I transmembrane protein belonging to the B7 family, which is encoded by the *CD274* gene on human chromosome 9 (4, 13). PD-L1 is ubiquitously expressed on immune cells, including T lymphocytes, B lymphocytes, and natural killer cells, as well as epithelial cells, vascular endothelial cells, APCs, multiple tumor cells, and tumor-infiltrating cells (4). The overexpression of PD-L1 on tumor cells is induced by genetic alterations (innate expression) and stimulation by interferon gamma (IFN-γ) released from effector T cells, including CD8+ T cells (acquired expression) (13, 29). PD-L1 expression also occurs in immune-privileged sites such as the eye and placenta, in which PD-L1 is overexpressed from the fourth month of gestation (14). The interaction of PD-1 and PD-L1 suppresses T cell receptor-mediated cytotoxicity and CD8+ T cell proliferation, which negatively regulate the adaptive antitumor immune response, avoid the killing effect on tumor cells, and evade immune surveillance (11, 16). Significantly, recent work demonstrated that the PD-L1:B7-1 ligand–ligand cis-interaction alters trans-interactions with other immune checkpoints, providing new insights into mechanisms of current pathways and immunotherapies (30, 31) (**Figure 3**). B7-1 (also called CD80) is a type I transmembrane protein that exists as a monomer and homodimer, and also belongs to the B7 family. B7-1 expressed on APCs binds to CD28 and CTLA-4 on T cells to provide co-stimulatory and co-inhibitory signals, respectively (32). Intriguingly, when PD-L1 is bound to B7-1 in cis, PD-L1 cannot engage PD-1 (30, 31). Meanwhile, the PD-L1:B7-1 cis-interaction disrupts the B7-1 homodimer and decreases its avidity to CTLA-4, thereby reducing B7-1 transendocytosis (31, 33). B7-1 binding to PD-L1 does not prevent its interaction with CD28, which consequently can form a trimeric complex (31). Nevertheless, the reports concerning the effect of the PD-L1:B7-1 cis-heterodimer on the B7-1:CD28 interaction are inconsistent (30, 33, 34). Moreover, the relative levels of PD-L1 and B7-1 will influence the outcome (31).

### 2.3 PD-L2

Programmed cell death ligand 2 (PD-L2, also known as B7-DC or CD273), a member of the B7 family of ligands, is also a type I transmembrane protein consisting of 270 amino acid residues, encoded by the *PDCD1LG2* gene (35, 36). Studies of PD-L2 as a therapeutic target and predictive biomarker are relatively few in comparison with PD-1 and PD-L1. Although PD-L2 and PD-L1 share the same receptor, PD-1, and have 37% sequence homology, they have differences in affinity and expression in various tissues (36). Studies have shown that PD-L2 had a 2 to 6-fold higher binding affinity to PD-1 than PD-L1 (35, 37, 38). PD-L2 is mainly present on APCs, such as macrophages and dendritic cells, and its expression can be induced in other immune and non-immune cells by various microenvironmental stimuli, especially Th2-associated cytokines (37), whereas PD-L1 is expressed on both immune cells and non-immune cells (36). At present, PD-L2 expression has been detected in patients with various malignancies and might predict worse prognosis (36–38). PD-L2 expression in human tumor samples generally correlates with that of PD-L1; however, PD-L2 expression was also present in the absence of PD-L1 in subsets of patient samples (39). Additionally, the role of PD-L2 is highlighted in studies of allergy and tolerance, and the second binding partner of PD­L2, repulsive guidance molecule b (RGMb), was discovered (31, 40, 41). RGMb is a glycosylphosphatidylinositol (GPI)-anchored protein and is one of three members of the repulsive guidance molecule family (RGMa/b/c) (31). RGMb also serves as a co-receptor for bone morphogenetic proteins 2 and 4 (BMP2 and BMP4) and neogenin, resulting in a supercomplex of BMP-BMPR-RGMb-neogenin in cis, and PD-L2 may bind in trans with the RGMb supercomplex to regulate downstream pathways (31, 40, 42) (**Figure 3**). The functional role of PD-L2 in this supercomplex requires further study.

## 3 Tumor immunity

Tumor cells exert immune escape and subsequently obtain unlimited proliferation ability because of the abnormal immune surveillance mediated by immune checkpoints. PD-1 and its ligands play a vital role in inhibiting immune responses and promoting self-tolerance by modulating the activity of T-cells, inhibiting cytokine secretion, and inducing apoptosis (11, 43). Inhibitors targeting the PD-1 pathway can rescue T cells from an exhausted state and revive the immune response against cancer cells (44, 45). Based on these observations, the relationship between tumors and PD-1/PD-L1/PD-L2 has been studied widely, and PD-1/PD-L1-targeted inhibitors, as cancer immunotherapy, have been developed (11, 13, 17, 29, 44–46). Various pathways modulate the PD-1/PD-L1 axis in tumorigenesis, including the PI3K/AKT pathway, the MAPK pathway, the JAK/STAT pathway, the WNT pathway, the NF-κB pathway, and the Hedgehog (Hh) pathway (13). To date, several monoclonal antibodies targeting the PD-1/PD-L1 signaling pathway have obtained first- and later-line US Food and Drug Administration (FDA) approval in various solid and hematological malignancies, including non-small cell lung cancer, melanoma, renal cell carcinoma, urothelial carcinoma, gastric and gastroesophageal junction adenocarcinoma, head and neck squamous cell carcinoma, and others, in which response rates range from 15-30% (in most solid tumors) to 45–60% (in melanoma and microsatellite instability-high tumors) (44, 47). Nivolumab (a fully human IgG4-blocking monoclonal antibody (mAb) against PD-1) was applied to the first-in-human trial of patients with advanced metastatic melanoma, colorectal cancer, castration-resistant prostate cancer, non-small cell lung cancer, and renal cell carcinoma in 2010, and first gained approval from the FDA to treat melanoma in 2014 (48–50). At present, numerous anti-PD-1 antibodies (nivolumab, pembrolizumab, cemiplimab, sintilimab, camrelizumab, toripalimab, tislelizumab, zimberelimab, prolgolimab, and dostarlimab) and anti-PD-L1 antibodies (atezolizumab, durvalumab, and avelumab) have been approved for various types of cancers (49) (**Table 1**). Approved anti-PD-1 and anti-PD-L1 antibodies differ in their molecular targets, epitope binding, affinity, structure, and pharmacokinetic characteristics (51). Based on these differences, it is possible that the efficacy and safety might vary among different anti-PD-1 and anti-PD-L1 agents (51). Currently, most immunotherapies targeting the PD-1 axis are antibody-based drugs. Bispecific antibodies, such as those co-targeting PD-1 and PD-L1 and those co-targeting PD-1 and CTLA-4, show enhanced treatment effect in anti-PD-1 resistant tumors (52–54). Apart from antibodies, many peptides against PD-1 and PD-1 and small molecular inhibitors that disrupt the PD-L1/PD-1 interaction and the PD-1/SHP-2 interaction, degrade PD-L1, or inhibit PD-1/PD-L1 expression at the mRNA level have been studied (52). Post-translational modification (PTM), such as phosphorylation, glycosylation, ubiquitination, and palmitoylation have been reported to modulate the function or homeostasis of PD-1 or PD-L1, which broadened the strategies for drug design (52). In addition, PD-1/PD-L1 blockade therapy combined with chemotherapy, radiotherapy, angiogenesis inhibitors, other immune checkpoint inhibitors, agonists of co-stimulatory molecules, stimulators of interferon gene agonists, fecal microbiota transplantation, epigenetic modulators, or metabolic modulators, have superior antitumor efficacies and higher response rates than conventional treatment (49).

**Table 1:** Anti-PD-1/PD-L1 antibodies and their indications.

Drug	Therapeutic target	Indication
Nivolumab	PD-1	Non-small cell lung cancer (NSCLC); Squamous cell carcinoma of head and neck; Gastric cancer; Malignant pleural mesothelioma; Esophageal cancer
Pembrolizumab	PD-1	Melanoma; NSCLC; Esophageal cancer; Squamous cell carcinoma of head and neck; Colorectal carcinoma; Hepatocellular carcinoma; Breast cancer
Toripalimab	PD-1	Melanoma; Nasopharyngeal carcinoma; Urothelial carcinoma; Esophageal cancer
Sintilimab	PD-1	Lymphoma; NSCLC; Hepatocellular carcinoma; Esophageal squamous cell carcinoma; Gastric cancer
Camrelizumab	PD-1	Lymphoma; Hepatocellular carcinoma; Esophageal cancer; NSCLC; Nasopharyngeal carcinoma
Tislelizumab	PD-1	Lymphoma; Urothelial carcinoma; NSCLC; Hepatocellular carcinoma; Esophageal cancer; Nasopharyngeal carcinoma; Microsatellite instability-high (MSI-H) or mismatch-repair deficient (dMMR) solid tumors
Penpulimab	PD-1	lymphoma
Zimberelimab	PD-1	lymphoma
Serplulimab	PD-1	MSI-H or dMMR solid tumors; NSCLC
Pucotenlimab	PD-1	MSI-H or dMMR solid tumors; Melanoma
Durvalumab	PD-L1	NSCLC; Small cell lung cancer (SCLC)
Atezolizumab	PD-L1	NSCLC; SCLC; Hepatocellular carcinoma
Envafolimab	PD-L1	MSI-H or dMMR solid tumors
Sugemalimab	PD-L1	NSCLC
Cadonilimab	PD-1/PD-L1	Cervical carcinoma

## 4 Autoimmunity

In autoimmune diseases, abnormal immune responses to self-antigens induce damage the body’s own tissues. The PD-1 pathway, as an inhibitory signal, controls the induction and maintenance of tolerance to self-antigens in the context of autoimmunity (55–58). Numerous pieces of evidence show that the PD-1 axis plays an significant role in autoimmune disorders, including inflammatory bowel disease (IBD), systemic lupus erythematosus (SLE), Type 1 diabetes (T1D), systemic vasculitis, myositis, autoimmune encephalomyelitis, autoimmune hepatitis, Behcet’s disease, myasthenia gravis, autoimmune uveitis, Sjogren’s syndrome, and ankylosing spondylitis (14, 57–61). Intestinal epithelial cells from patients with IBD were observed to overexpress PD-L1 and PD-L2, which might result in the regulation of immune responses against chronic inflammation and then prevent progressive and acute inflammation during the disease course (62, 63). The overexpression of PD-1, PD-L1 and PD-L2 on various immune cells of patients with SLE has been reported, and PD-1 receptors and their ligands have been identified to be involved in two key pathways, the toll-like receptor (TLR) pathway and the type I interferon (IFN-1) pathway through activation of NF-κB and/or STAT1 in the pathogenesis and development of SLE (14, 64). A murine model demonstrated significantly reduced severity of insulitis and delayed diabetes progression, associated with a reduction of spontaneous diabetes incidence in transgenic mice with the upregulation of PD-L1 (65). Restoring the PD-1/PD-L1 function could represent a valid strategy to treat T1D at different stages, including regulating β cell autoimmunity and preventing T1D in individuals that are genetically at-risk or are autoantibody positive; promoting immune tolerance and preserving residual β cell mass in patients with new onset T1D; and reducing alloreactive responses and favoring the survival of transplanted islets in patients with established T1D disease (66). Hakroush et al. observed that loss of tubulointerstitial PD-1 correlated with active antineutrophil cytoplasmic antibody (ANCA)-associated renal vasculitis, and PD-1 was associated with decreased local synthesis of complement factor B (67). Co-culture approaches *in vitro* showed that monocytes from patients with ANCA-associated vasculitides displayed low expression of PD-L1 and a defective PD-L1 presentation upon activation, thus increasing the expression of PD-L1 might reduce the level of ANCA and improve disease activity (68). PD-1 expression on CD57+ and CD8+ cells increased early, fluctuated, and then increased again in later stages in patients with inclusion body myositis (IBM), and the expression of PD-L1 and PD-L2 were observed on adjacent cells, including muscle fibers (61). Dalakas proved that the formation of immunological synapses between autoinvasive T cells and muscle fibers was strengthened by the upregulation of PD-L1 in polymyositis (PM) and sporadic IBM (69). Recently, a homozygous loss-of-function mutation in *PDCD1* was identified in a child manifesting with multiorgan autoimmunity and *Mycobacterium tuberculosis* infection, which suggested inherited complete PD-1 deficiency (70). Several different approaches have been developed to enforce PD-1/PD-L1 stimulation, such as PD-L1–Fc fusion, PD-1 stimulatory agents, and anti-PD-1 agonist monoclonal antibodies (mAbs) (34, 55, 71–73). Administration of PD-L1-Fc significantly ameliorated inflammatory colitis in murine models, which suggested that PD-1-mediated inhibitory signals might represent a novel target (73). A previous investigation of lupus-like syndrome in mice showed that PD-L1 was overexpressed on renal proximal tubular epithelial cells after Ad-PD-L1 injection, and the frequency of proteinuria was lower, serum levels of anti-dsDNA IgG decreased, and renal pathology improved (74). Suzuki et al. identified PD-1 agonists that inhibit T cells by triggering immunosuppressive signaling in murine disease models with acute graft versus host disease (aGVHD) and colitis, and indicated their clinical potential to treat autoimmune diseases (55). Sugiura et al. demonstrated that the removal of PD-1 restriction is effective in alleviating autoimmune disease symptoms in murine models with arthritis, multiple sclerosis and Sjögren’s syndrome by targeting the cis-PD-L1–CD80 duplex (34). CD80 binding to PD-L1 inhibits its interaction with PD-1; therefore, the blockade of CD80–PD-L1 binding attenuates PD-L1–PD-1 binding and abrogates PD-1 function (34).

## 5 Infection immunity

Studies regarding the PD-1 pathway in the context of viral, bacterial, and parasitic infections are accumulating (46, 59, 75–78). During infection, peptide antigens from microbes are presented on MHC complexes to naive T cells by APCs. CD4+ T cells recognize MHC class II molecules to modulate the immune response against extracellular pathogens by coordinating B cells and activating innate effector cells; CD8+ T cells recognize MHC class I molecules and function as cytotoxic cells (9). The PD-1 pathway serves as a coinhibitory signaling pathway to regulate the activation and function of T cells at numerous points, and PD-1 blockade might have a potential therapeutic effect on infectious diseases, especially chronic viral infection (10, 75–78). During the course of a chronic viral infection, the continuous burden of viral antigens leads to persistent stimulation of antigen-specific T cells, which causes T cell exhaustion (9, 46). PD-1 expression on virus-specific T cells has been documented in infections with lymphocyte choriomeningitis virus (LCMV), human immunodeficiency virus (HIV), hepatitis B virus (HBV), hepatitis C virus (HCV), and coronavirus disease 2019 (COVID-19) (10, 78). In chronic infection with HBV, PD-1 expression on HBV-specific T cells is increased and might serve as a biomarker for liver damage (9, 79). Upregulation of PD-1 in regulatory T cells (Tregs) was also found in patients with chronic HCV infection, and the observation that blockade of PD-1 improved Treg function suggested that PD1 acts as negative regulator of Tregs in this setting (80). As previously reported, the PD-1 antibody nivolumab helped to treat seven patients with relapsed/refractory Epstein-Barr virus (EBV)-associated hemophagocytic lymphohistocytosis and 71.4% of them reached a clinical complete response without relapse (81). You et al. reported a patient with adult-onset chronic active Epstein-Barr virus infection (CAEBV) after allogeneic hematopoietic stem cell transplantation (allo-HSCT) who was treated with salvage PD-1 antibody sintilimab, in whom EBV-DNA was ultimately undetectable (82). PD-1 signaling has also been examined in the context of numerous bacterial infections, such as *Mycobacterium tuberculosis* (Mbt) and *Helicobacter pylori*. In Mbt infection, the enhanced expression of PD-1 and PD-L1 was observed in patients with tubercle bacillus and in Mbt-treated mice (10, 83). However, there are reports of Mbt reactivation among patients with cancer being treated with PD-1 blockade (84). PD-1 and PD-L1 expression levels were also found to be higher in patients infected with *Helicobacter pylori*, which might be associated with the increased frequency of gastric cancer (9). Parasitic infections are associated with immune evasion by increasing anti-inflammatory molecules and the expression of coinhibitory receptors and their ligands, which often present as chronic infections (9). The PD-1 pathway has been described during infection with a number of parasitic protozoans, including *Toxoplasma gondii*, *Leishmania major*, *Plasmodium falciparum*, *Plasmodium berghei*, and *Babesia microti* (9, 85, 86). Blocking the PD-1 pathway in mice infected with *Plasmodium berghei* ANKA induced strong natural and acquired immune responses, and enhanced immune memory against the parasite (86). In addition, the administration of PDL1-IgG1Fc in a mouse model of experimental cerebral malaria showed a protective effect based on the maintenance of immune microenvironment homeostasis in the brain via repressing over-reactive CD8+ T cell responses (85). PD-1 might have contributed to the establishment of the mutual existence of the host and the pathogens. While PD-1 blockade might enhance pathogenic microbe clearance in patients with acute and chronic infections, there is a risk that reactivation of subsets of exhausted T cells might increase tissue immunopathology, leading to immune-related adverse events. Numerous studies have linked PD-1 and its ligands to altered immune cell activity in sepsis (87–89). Ruan et al. found that sepsis might induce an immunosuppressive state, resulting in myeloid derived suppressor cell (MDSC) expansion, and upregulation of PD-L1 on MDSCs is linked to increasing PD-1 on T cells and the induction of T cell apoptosis (88). Patients with sepsis, especially severe sepsis and septic shock, had obviously higher expression levels of PD-1 on CD4+ or CD8+ T cells, PD-L1 on monocytes, sPD-1, and sPD-L1 compared with patients with non-septic infections, non-infectious inflammation, and a healthy control group (89). Currently, studies regarding anti-PD-1/PD-L1 antibodies for the treatment of sepsis in animal models or in patients with sepsis have been reported (51, 90).

## 6 Transplantation immunity

In allogenic transplantation, the transplant expresses foreign antigens, increasing the risk of rejection. T cell activation by immune allorecognition is a major contributing factor towards triggering organ rejection (91). Immune checkpoints are crucial regulators of the immune system for self-tolerance and the prevention of rejection in the context of transplantation. To date, PD-1 and its ligands have been reported to play a significant role in the balance between reactive T cells targeting the organ and tolerogenic Tregs (59, 91–93). Upregulation of PD-1, PD-L1, and PD-L2 in a murine model of cardiac transplantation during the process of allogeneic rejection was observed compared with that in syngeneic transplants and normal tissues (94). The application of PD-L1.Ig markedly enhanced allograft tolerance and prolonged transplant acceptance in mice, which in some cases led to permanent engraftment and accompanied reduced intragraft expression of IFN-γ and IFN-γ-induced chemokines (94). In samples of human transplanted hearts, acute cellular rejection was demonstrated to be associated with decreased PD-L1 expression in the lymphocyte cell population compared with PD-1 expression (95). The upregulation of PD-L1 expression in both dendritic cells and allografts via FK506-binding protein (FKBP) 51 in FK506-mediated immunosuppression was observed in a murine heart transplantation model (96). The PD-1 pathway has been reported to modulate rejection of renal transplants in animal experiments and clinical studies. Most T cells expressed PD-1 (over 90% of CD8+ T cells and about 75% of CD4+ T cells) during the initial response to murine kidney transplants. Administration of a blocking antibody to PD-L1 increased T cell infiltrates and urinary Lipocalin 2 (LCN2), causing terminal acute rejection (97). The expression of PD-L1, PD-L2, and PD-1 mRNA and protein was upregulated in biopsies of patients with renal allograft rejection compared with the respective levels found in the pretransplant biopsies (98). High PD-1 expression in several T cell subsets predicts a higher rate of rejection in the clinic (99). The upregulation of PD-L1 on proximal tubular epithelial cells in patients with acute allograft rejection might reduce T-cell-mediated injury by inhibiting the proliferation of CD4+ T cells and cytokine production by CD8+ T cells (98). Luo et al. developed a membrane-anchored-protein PD-L1 (map-PD-L1), which was effectively anchored onto the surface of rat glomerular endothelial cells and binds PD-1 (93). They found that map-PD-L1 could reduce T cell graft infiltration and increase intragraft Treg infiltration, suggesting a long-term effect in allograft protection in kidney transplantation models (93). In addition, several studies regarding the PD-1 pathway in other transplantations, including skin, liver, islet, and allogeneic hematopoietic stem cells, have also been reported (91, 100–102). Mechanistically, PD-1 and its ligands, PD-L1 and PD-L2, constitute an inhibitory regulatory pathway with potential therapeutic use in transplanted organs undergoing allograft rejection (92).

## 7 Allergy

Allergic diseases, such as asthma, rhinoconjunctivitis, atopic dermatitis, food and drug allergy, are characterized by pathological and overactive immune responses against harmless antigens, especially type 2 immune responses. The allergic inflammatory process involves different cell types that release a range of inflammatory mediators and cytokines, including IgE-dependent activation and increased CD4+ T helper type 2 (Th2) lymphocytes (103). PD-1 and its ligands play an essential role in regulating T cell activation and function. These immune checkpoint molecules balance the immune response, preventing the accumulation of self-reactive T cells. Consequently, PD-1/PD-L1 or PD-L2 signaling in allergy has been studied (12, 103, 104). Interestingly, PD-L1 and PD-L2 might have opposing roles in the pathogenesis of asthma (12, 105). PD-L2 downregulates IL-4 and upregulates IFN-γ to decrease airway hyperreactivity (AHR), whereas PD-L1 upregulates IL-4 and downregulates IFN-γ (103). Mouse experiments demonstrated that PD-L2 deficiency results in increased AHR and lung inflammation (105). However, a lack of PD-L1 leads to reduced levels of AHR, and minimal inflammation and pulmonary mucous secretion in asthma pathogenesis (12). A mouse model of allergic asthma displayed that PD-1/PD-L1 blockade enhanced AHR by developing a concomitant Th17 immune response (106). Recent research showed that normal resting lung interstitial macrophages and alveolar epithelial cells express high levels of *RGMB* mRNA, whereas lung dendritic cells express PD-L2 (42). The RGMb : PD-L2 interaction has been demonstrated to promote the development of respiratory tolerance (42). Evidence suggested that the expression levels of PD-1 and its ligands on the surface of immune cells in the nasal mucosa are higher in patients with allergic rhinitis than in non-allergic patients (103). Moreover, increased soluble PD-L1 has been detected in the serum of patients with allergic rhinitis, and soluble PD-L1 showed a significant negative association with disease severity, symptom intensity, and eosinophil counts (107). In a murine model of active cutaneous anaphylaxis, anti-PD-L1 blockade during the sensitization phase led to a reduction in specific IgE and IgG1 levels, decreased allergic reaction intensity at the active cutaneous anaphylaxis site, and less mast cell degranulation in the tissue; however, this did not occur during the challenge phase (108). PD-L1-deficient mice had more severe changes in ear thickness in Th1-and Th17-type immunity models than PD-L2-deficient mice; For the Th2 type model, PD-L2-deficient mice had more severe changes in ear thickness, which suggested that PD-L1 has lesser role in Th1 and Th17 type immunity, whereas PD-L2 is predominant in Th2 type immunity (109). Lama et al. reported that anti-PD-1 treatment or genetic deficiency of PD-1 in CD4+ T cells inhibited the production of peanut-specific IgE and increased the levels of IgG in mice, which demonstrated that blockade of the pathway between PD-1 and its ligand is protective against allergic immune responses (104).

## 8 Immune privilege

The immune-privileged microenvironment represents a special immunological condition, where foreign antigens can be tolerated without inducing excessive immune responses. The anterior chamber of the eyes, testis, and the pregnant uterus are all regarded as physiological immune-privileged sites in humans. As previously mentioned, PD-1 signaling plays an important role in controlling autoimmunity and inducing immune tolerance (46, 59). Consequently, this inhibitory pathway has been studied in the maintenance of immune privilege (110, 111). Yang et al. (112) found that PD-L1 was expressed constitutively in human ocular cell lines and was significantly upregulated in inflamed ocular tissues compared with that in normal eyes. Moreover, IFN-γ, TNF-α, and IL-5 production by activated T cells cocultured with ocular cells was significantly enhanced in the presence of an anti-PD-L1 blocking antibody (112). Animal experiments demonstrated that PD-1 and PD-L1 were present in the testicular tissue of adult mice (113). PD-1 was mainly localized to the germ cells and was dependent on the developmental stage of the mouse, suggesting that it might play a role in spermiogenesis (113). PD-L1 was constitutively expressed in Sertoli cells, which could secrete soluble PD-L1 into the testicular interstitial space and thus might be involved in testicular immune privilege (113). Recent data have shown that PD-L1 is overexpressed in testicular germ cell tumors, but is not expressed in normal human testicular tissue, suggesting the potential for PD-1/PD-L1 as therapeutic targets in testicular germ cell tumors (114). The PD-1/PD-L1 pathway plays a vital role in the process of allowing pregnancy by suppressing the maternal immune response to paternally inherited alloantigens (110). PD-L1 expression increases during the progression of pregnancy (59, 110). Decreased mRNA and protein levels of PD-L1 in placental villi were observed in women with recurrent miscarriage in comparison with early normal pregnancy (115). Blockade of PD-L1 signaling during murine pregnancy resulted in higher rates of premature terminated pregnancies and decreased litter sizes (116). Trophoblastic cells create a tolerogenic fetal-maternal interface by upregulating PD-L1 in syncytiotrophoblasts and intermediate trophoblasts, and trophoblastic tumors might also use PD-L1 expression to evade the host immune response, thereby promoting their survival (117). B7-H3 and VISTA are also observed to be highly expressed in gestational trophoblastic neoplasia (GTN), and might be potential immunotherapeutic targets for GTN treatment (118).

## 9 Immune-related adverse events related to anti-PD-1 and anti-PD-L1 drugs

With the increasing therapeutic use of anti-PD-1/PD-L1 immune-checkpoint antibodies in the clinic, more immune-related adverse events (irAEs) involving the skin, endocrine glands, gastrointestinal tract, lungs, liver, heart, and blood have been reported (119–123). The application of immune checkpoint inhibitors (ICIs) destroys the protection of the autoimmune response, enhances the activity of T cells against antigens presented in tumors and healthy tissues, and increases the level of pre-existing autoantibodies and inflammatory cytokines, leading to a series of irAEs, which are discrete and nonspecific (124). Patients responding to ICIs are thought to have a greater likelihood of autoimmune toxicities and a higher risk of irAEs because of their more treatment-responsive immune system and cross-reactivity between the tumor and host tissue (47). Differences regarding the risk of irAEs might exist between the different types of anti-PD-1 and anti-PD-L1 antibodies (125, 126). Anti-PD-1 antibodies might induce more irAEs than anti-PD-L1 antibodies because inhibitors targeting PD-1 also block the binding of PD-L2, which might generate inhibitory signals affecting the immune response (125). A trial-level meta-analysis showed a lower risk of overall grade ≥ 3 irAEs with anti-PD-L1 antibodies compared with that from anti-PD-1 antibodies (126). Furthermore, lower risks of overall any-grade irAEs and grade ≥ 3 irAEs were observed for atezolizumab and avelumab versus pembrolizumab, respectively (126). In addition, different tumor types might drive different irAEs. Dermatitis and arthritis were more common in patients with melanoma than in those with renal cell carcinoma, while pneumonia and dyspnea were less common in patients with melanoma (124).

A broad spectrum of irAEs affects almost all tissues and organs, with a variety of clinical presentations and diagnostic considerations (119). The incidence and mortality of irAEs have been reported as 15–90% and 0.3–1.3%, respectively, in clinical studies (127). Adverse cutaneous toxicities are the most prevalent irAEs and mainly manifest in the form of maculopapular rash and pruritus (119). Dermatological toxicities are usually mild and rarely life-threatening, including Steven-Johnson syndrome and toxic epidermal necrolysis (128). Immune-related endocrine events are very common, such as hyperthyroidism, hypothyroidism, diabetes mellitus, and hypophysitis, which can be treated by exogenous administration of the missing hormone (129). ICI-induced gastrointestinal toxicities are one of the most common irAEs affecting patients, involving the oral mucosa to the rectum, with mild to life-threatening symptoms. The main clinical manifestations are diarrhea, colitis, and inflammatory bowel disease, and most occur after 2–3 cycles of ICI treatment (124). In patients treated with anti-PD-1 antibodies, 6.0–16.0% of them reported gastrointestinal irAEs (130). Pulmonary toxicities described in irAEs include pneumonitis, sarcoidosis, pleural effusions, and reactive airway disease. The incidence of checkpoint-inhibitor pneumonitis in patients with melanoma receiving anti-PD-1 monotherapy and combination therapy was 3.8% and 9.6%, respectively (129). Hepatitis is a relatively common irAE and presents as an asymptomatic increase of aspartate transaminase, alanine transaminase, and total bilirubin (130). Cardiovascular irAEs are relatively uncommon, but are very dangerous, with considerable mortality. Myocarditis is the most commonly documented cardiovascular complication of ICIs and might manifested as fatigue, chest pain, acute heart failure, cardiogenic shock, arrhythmias, or sudden death (127). The hematological adverse events affected by ICIs have reported frequencies of 3.6% for all grades and 0.7% for grades III–IV, and included immune thrombocytopenia, pancytopenia, aplastic anemia, neutropenia, hemolytic anemia, bicytopenia, pure red cell aplasia, and cytokine release syndrome with hemophagocytic syndrome, in which their frequency was higher under anti-PD-1 and anti-PD-L1 therapy than under anti-CTLA-4 therapy (131). Prompt recognition and intervention for immune-related toxicities are required to optimize clinical outcomes.

## 10 Conclusion

Significant advances have been made in immunotherapy in the last decade because of our increased understanding of the biological consequences of immune checkpoint molecules. Immune checkpoint blockade can recover T cell activation and might provide novel therapeutic approaches for immune-associated disorders. In this review, we focused on the role of PD-1 signaling in health and immune-related diseases. Fundamental research concerning the PD-1 pathway in tumor immunity, autoimmunity, infection immunity, transplantation immunity, allergy, and immune privilege, has expanded our knowledge of immune regulation and supports the development of drugs that modulate immunity. In addition, irAEs are important in the successful application of the immune checkpoint blockers targeting PD-1 receptor and its ligands.

## Author contributions

R-YC researched and drafted the initial manuscript. QL helped to designed and revised this review. X-ZL commented on the manuscript. YZ, Y-YS, Q-YX, H-YT, N-XC, LJ, X-MD, and W-QC were involved in searching literatures and editing the manuscript. All authors contributed to the article and approved the submitted version.

## Glossary

**Table:**

PD-1	programmed cell death 1
PD-L1	programmed cell death 1 ligand 1
PD-L2	programmed cell death 1 ligand 2
MHC	major histocompatibility complex
TCR	T cell receptor
APCs	antigen-presenting cells
CTLA-4	cytotoxic T lymphocyte-associated antigen-4
CD28	cluster of differentiation 28
CD80	cluster of differentiation 80
Gal-9	galectin-9
TIM-3	T cell immunoglobulin and mucin domain 3
irAEs	immune-related adverse events
Ig	immunoglobulin
ITIM	immunoreceptor tyrosine-based inhibitory motif
ITSM	immunoreceptor tyrosine-based switch motif
ICOS	inducible co-stimulatory molecule
NFAT	nuclear factor of activated T cells
FOX	Forkhead box protein
IRF9	interferon regulatory factor 9
CTLs	cytotoxic T lymphocytes
PI3K	phosphoinositide 3-kinase
PDK1	phosphoinositide-dependent kinase 1
mTOR	mammalian target of rapamycin
MAPKK	mitogen-activated protein kinase kinase
ERK	extracellular-signal-regulated kinase
JAKs	Janus kinases
STAT	signal transducers and activators of transcription
VISTA	V-domain Ig suppressor of T cell activation
CRD	carbohydrate-recognition domain
PD-1H	programmed cell death 1 homolog
PSGL-1	P-selectin glycoprotein ligand 1
VSIG3	V-set and Ig domain-containing 3
IFN-γ	interferon gamma
RGMb	repulsive guidance molecule b
GPI	glycosylphosphatidylinositol
BMP	bone morphogenetic proteins
Hh	Hedgehog
FDA	Food and Drug Administration
PTM	Post-transcription modification
IBD	inflammatory bowel disease
SLE	systemic lupus erythematosus
T1D	Type 1 diabetes
TLR	toll-like receptor
IFN-1	type I interferon
ANCA	antineutrophil cytoplasmic antibody
IBM	inclusion body myositis
PM	polymyositis
mAbs	monoclonal antibodies
aGVHD	acute graft versus host disease
LCMV	lymphocyte choriomeningitis virus
HIV	human immunodefficiency virus
HBV	hepatitis B virus
HCV	hepatitis C virus
COVID-19	coronavirus disease 2019
Tregs	regulatory T cells
EBV	Epstein-Barr virus
CAEBV	chronic active Epstein-Barr virus infection
allo-HSCT	allogeneic hematopoietic stem cell transplantation
Mbt	Mycobacterium tuberculosis
MDSCs	myeloid derived suppressor cells
FKBP	FK506-binding protein
LCN2	Lipocalin 2
map-PD-L1	membrane-anchored-protein PD-L1
Th2	T helper type 2
AHR	airway hyperreactivity
GTN	gestational trophoblastic neoplasia
ICIs	immune checkpoint inhibitors.
